# NR4A2 may be a potential diagnostic biomarker for myocardial infarction: A comprehensive bioinformatics analysis and experimental validation

**DOI:** 10.3389/fimmu.2022.1061800

**Published:** 2022-12-22

**Authors:** Dongsheng Wei, Jiajie Qi, Yuxuan Wang, Luzhen Li, Guanlin Yang, Xinyong He, Zhe Zhang

**Affiliations:** ^1^ Graduate Academy, Liaoning University of Traditional Chinese Medicine, Shenyang, Liaoning, China; ^2^ Key Laboratory of Ministry of Education for Traditional Chinese Medicine Viscera-State Theory and Applications, Liaoning University of Traditional Chinese Medicine, Shenyang, Liaoning, China; ^3^ College of Medical Laboratory, Liaoning University of Traditional Chinese Medicine, Shenyang, Liaoning, China

**Keywords:** myocardial infarction, nomogram, single-cell sequencing (scRNA-seq), immune infiltration, diagnostic model, bioinformatics

## Abstract

**Background:**

Myocardial infarction is a well-established severe consequence of coronary artery disease. However, the lack of effective early biomarkers accounts for the lag time before clinical diagnosis of myocardial infarction. The present study aimed to predict critical genes for the diagnosis of MI by immune infiltration analysis and establish a nomogram.

**Methods:**

Gene microarray data were downloaded from Gene Expression Omnibus (GEO). Differential expression analysis, single-cell sequencing, and disease ontology (DO) enrichment analysis were performed to determine the distribution of Differentially Expressed Genes (DEGs) in cell subpopulations and their correlation with MI. Next, the level of infiltration of 16 immune cells and immune functions and their hub genes were analyzed using a Single-sample Gene Set Enrichment Analysis (ssGSEA). In addition, the accuracy of critical markers for the diagnosis of MI was subsequently assessed using receiver operating characteristic curves (ROC). One datasets were used to test the accuracy of the model. Finally, the genes with the most diagnostic value for MI were screened and experimentally validated.

**Results:**

335 DEGs were identified in GSE66360, including 280 upregulated and 55 downregulated genes. Single-cell sequencing results demonstrated that DEGs were mainly distributed in endothelial cells. DO enrichment analysis suggested that DEGs were highly correlated with MI. In the MI population, macrophages, neutrophils, CCR, and Parainflammation were significantly upregulated compared to the average population. NR4A2 was identified as the gene with the most significant diagnostic value in the immune scoring and diagnostic model. 191 possible drugs for the treatment of myocardial infarction were identified by drug prediction analysis. Finally, our results were validated by Real-time Quantitativepolymerase chain reaction and Western Blot of animal samples.

**Conclusion:**

Our comprehensive in silico analysis revealed that NR4A2 has huge prospects for application in diagnosing patients with MI.

## Introduction

Myocardial infarction (MI) results from severe coronary artery disease, with high morbidity and mortality rates worldwide, according to the World Health Organization (WHO) (1). Myocardial infarction, ischemic cardiomyopathy, and arrhythmia are regarded as an aggravation of coronary artery disease (CAD), a pathological evolution characterized by atherosclerotic plaque accumulation, inflammation, and endothelial dysfunction in the epicardial arteries (2). Acute coronary syndrome causes thrombosis and acute ischemia due to unstable atherosclerotic plaque rupture in the coronary artery. Although the past decade has witnessed significant progress achieved in understanding the pathogenesis of myocardial infarction, the exact mechanisms remain unclear (3). The development of single-cell sequencing technology has allowed us to further understand the pathogenesis of myocardial infarction (4). In the present study, we used single-cell sequencing technology to identify the cell subpopulations associated with the pathogenesis of myocardial infarction, providing the foothold for future studies. The prognosis of myocardial infarction is closely related to the diagnosis, and improving the diagnostic accuracy of patients with myocardial infarction is essential to improve clinical outcomes and prognosis. Cardiovascular troponin, routinely used in clinical practice to diagnose MI and initiate personalized treatment, is an essential biomarker from a clinical perspective (5). However, cardiac troponin, the biomarker of choice, is associated with a significant lag time before increasing after MI. With the advancement of modern research, genetic tests can nowadays be performed in less than 15 minutes (6), reducing the time to clinical diagnosis of MI, but this first requires us to identify the most specific gene. Accordingly, this study sought to identify a diagnostic gene with high specificity. A rat study found that organismal immune genes were first detected within 30 minutes of myocardial infarction compared to other types of genes (7), highlighting the early diagnostic value of immune-related gene expression. Although some diagnostic markers of myocardial infarction have previously been documented by transcriptome analysis of immune cells and their function, their mechanism in myocardial infarction remains to be elucidated.

An increasing body of evidence highlights the importance of the immunological milieu in the development, progression, and instability of atherosclerotic plaques. Immunological infiltration has been utilized to examine the tumor immune milieu, identify diagnostic genes for distinct cancers, and predict suitable therapy and prognosis for patients (8). Other non-tumor inflammatory diseases, such as lupus nephritis, have also been widely applied for analyzing immune cell filtration (9). Considering the significance of immune infiltration in the pathophysiology of MI, establishing a model based on immune-related genes is critical for optimizing the diagnosis of MI.

Nomograms were first reported in clinical application in 1928. They are statistical tools for the graphic illustration of a posh mathematical formula (10, 11). Medical nomograms combine biological and clinical data, measuring the score for factors on a scale to visually display a statistical model that predicts the likelihood of clinical events for individual (12, 13). Compared to conventional staging, the convenient and concise operation of a nomogram, with increased accuracy, aids in precise clinical decision-making, accounting for their increased use by researchers and doctors.

To our knowledge, few studies have explored diagnostic genes, cell subpopulation distribution, and immune infiltration in patients with MI. We applied single-sample genomic enrichment analysis (ssGSEA), differential expression analysis, immune infiltration, and diagnostic modeling by constructing immune-related genes to reveal new diagnostic genes for MI. In addition, drug response prediction can help us identify potential drugs for MI treatment.

## Materials and methods

### Microarray data extracted from gene expression omnibus and data processing

**Figure 1:**
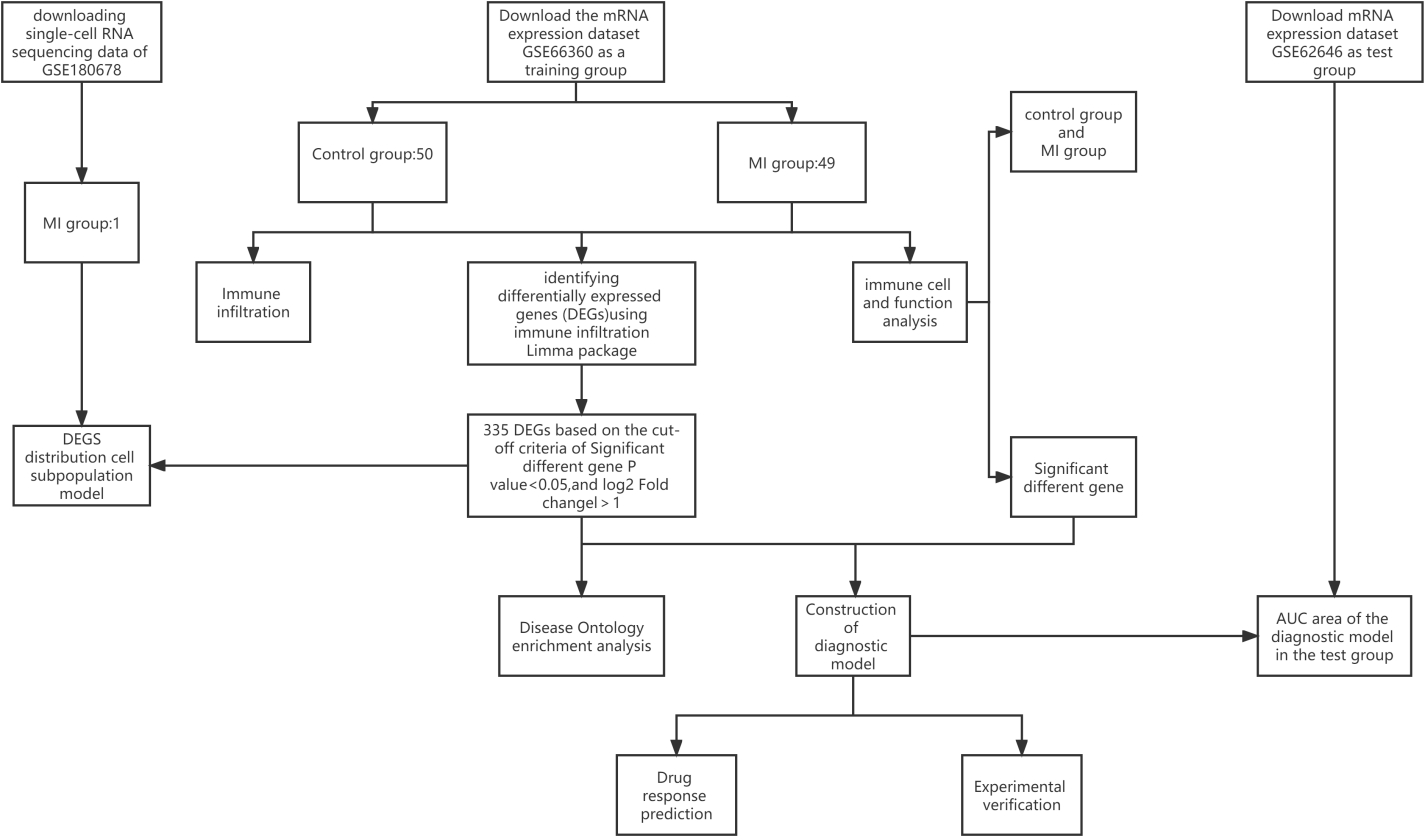
Flowchart of the current study.

A flowchart of the current study is illustrated in **Figure 1**. Dataset GSE66360 based on the Affymetrix platform (**Table 1**) was downloaded from the Gene Expression Omnibus database (14), consisting of 99 samples, including 49 MI samples and 50 normal samples. The test dataset GSE62646 contains 84 MI samples and 14 patients with coronary artery disease (CAD). The single-cell dataset GSE180678 was obtained from the GEO database, which contained one patient with ischemic cardiomyopathy (**Table 1**).

**Table 1:** Descriptive statistics.

Data number	Platform information	MI group	Control group	Species
GSE66360	GPL570	49	50	Homo sapien
GSE180678	GPL20301	1	0	Homo sapien
GSE62646	GPL6244	84	14	Homo sapien

### Characteristics of differentially expressed genes from dataset GSE66360

Data were preprocessed before performing the DEG analysis. The Affymetrix in R was utilized to improve the database *via* background calibration, normalization, and log2 transformation (15). We averaged the probe site values if a gene had more than one. According to the annotation files from the platform, the probe IDs were converted to gene symbols, and probes that did not correspond to the symbol of the gene were removed. Finally, the eligible DEGs were screening and electing by the linear models for microarray (LIMMA) data package; P-value < 0.05, and |log2 FC| > 1 (16).

### Single-cell sequencing of DEGs

First, we used Seurat (17) for data processing to screen reliable cell subpopulations from single-cell data. The expression of each gene must occur in at least three cells, with at least 200 genes expressed per cell. The percentages of mitochondria and rRNA were calculated by the PercentageFeatureSet function, ensuring that each cell expressed more than 500 genes and <4,000 genes with <30% mitochondrial content and at least 100 unique molecular identifiers (UMIs) per cell. The data were then normalized by log normalization and used to identify highly variable genes using the FindVariableFeature function. Principal Component Analysis (PCA) was used to scale down all genes using the ScaleData function. Next, subgroups of cells were determined using the FindNeighbors and FindCluster functions (set resolution = 0.1), and annotations were applied. The R package FindAllMarkers was used to identify markers for each cell subpopulation and the gene expression profile dataset was re-evaluated using the CiberSort (18) algorithm in the context of the expression of marker genes for each cell subpopulation to determine the composition of individual cell subpopulations in different samples in the expression profile. Finally, DEGs were combined with cell subpopulations to analyze the distribution and abundance of DEGs in cell subpopulations.

### Disease ontology enrichment analysis of DEGs

Differential genes are the basis of our subsequent analysis study, and whether DEGs are associated with MI determines the accuracy of our following study. Therefore, we performed DO enrichment analysis on differential genes to observe the degree of enrichment of DEGs in MI. We used the “cluster Profiler” package (19) and “DOSE” package (20) for DO enrichment analysis to analyze the enrichment degree of differential genes in MI and visualize the results on a bar chart (barplot) and a bubble plot (dotplot).

### Immune infiltration analysis

It has been established that ssGSEA can be used to quantify the correlative scale of immunological cells and functions from DEGs. Thus, ssGSEA was adopted to evaluate the correlation between a single sample gene of immune cells and functions based on the converged biological data (21). Then, the results of immune cells and immune function were visualized in a heat map using the “pheatmap” package (22). Finally, the R package “GGally” was utilized to visualize the correlation between 16 immune cells and 12 immune functions. A P-value < 0.05 was statistically significant.

### Analysis of immune score for DEGs between control and MI groups

The divergence in immune scores between normal and MI groups and the difference in immune cells and functions between the cohorts were assessed by data conversion and visualization using ggplot2 and reshape2 statistical packages by R. We further conducted a correlation analysis of significantly expressed genes between immune cells and function

Next, we selected the top five upregulated and downregulated genes among the most significant DEGs to clarify the correlation between MI-significant differential genes and immune cells and function. The top six genes mostly associated with immunity were screened as hub genes. Subsequently, the data were visualized using ggplot2 packages.

### Construction of immune-related gene model

The identified hub genes were used to establish a gene-disease diagnostic model to determine the accuracy of the genes mostly associated with immune cells and function for MI diagnosis. The goodness-of-fit between the actual and predictive values and the degree of the fitting was evaluated using a calibration plot and Spiegelhalter’s Z-test. In addition, the receiver operating characteristic curve (ROC), the area under the ROC curve (AUC), and Harrell’s concordance index (C-index) were utilized to appraise the nomogram’s discrimination ability. Finally, we compared the levels of hub gene expression between control participants and MI cases in GSE62646 to validate the accuracy of the model by the same analysis methods as above.

### Drug prediction

To expand the clinical value of the immune-related gene model, the Enrichr platform (https://maayanlab.cloud/Enrichr/) and the coremine platform (https://www.coremine.com) were used to predict the drug delivery of NR4A2 in the model. Drug predictions are identified through the Drug Signature Database (DSigDB) in Enrichr, a large portal with a large number of different genomic libraries that identify targeted drugs associated with NR4A2. A P-value < 0.05 was used to select drugs with potential clinical application.

### Experimental animal preparation

Twenty SD rats were divided into an experimental group (n=17) and a control group (n=3). After 7 days of adaptation, the rats underwent ligation of the anterior descending branch of the coronary artery after anesthesia on day 8. An electrocardiogram was performed after ligation to observe whether the ST segment of the rats showed evident elevation, and the ST-segment elevation indicated successful modeling. After the operation, the rats were fed for seven days. The rat’s abdominal aorta was severed on postoperative day 8 to induce massive hemorrhage and death, and the heart tissue was harvested after death. We divided 6 rats into two groups for quantitative real-time PCR (qPCR) and WB (MI=3, control=3). This study was approved by the Ethics Committee of the Affiliated Hospital of Liaoning University of Traditional Chinese Medicine (2022CS(DW)-006-01).

### Western blot

50 mg of tissue from the ischemic portion of the left ventricle of the experimental group and the control group were harvested for qPCR and WB experiments, cut in pre-chilled PBS, and centrifuged for 5 min. After the supernatant was removed, the tissue was homogenized by adding the corresponding lysate volume. Then the tissues were fully lysed at 4°C for 20 min and then centrifuged at 12 000 r/min for 20 min. BCA detected the protein concentration in the supernatant, and the remaining supernatant was mixed with protein loading buffer (5×Loading Buffer) at a ratio of 1:4 and boiled to denature the protein. After separation by protein gel electrophoresis, the proteins were transferred to PVDF membranes and blocked with 5% skim milk. The membranes were incubated with the primary antibody at 4°C at a dilution ratio of 1:5000 for NR4A2 and 1:5000 for GAPDH, incubated overnight, eluted three times with TBST for 10 min each, and 1:5000 HRP-labeled secondary antibodies (goat anti-mouse and goat anti-rabbit) were added, respectively The proteins were incubated for 2 h at room temperature, eluted three times with TBST for 10 min each time, developed by exposure with ECL chemiluminescent solution, photographed by Bio-RAD camera system and analyzed by Image Lab software for relative expression of proteins.

### Quantitative real-time PCR

50mg of experimental and control tissues were taken, added to 1mL Trizol, and homogenized on ice. Total RNA was extracted and cDNA synthesized. All primers were designed with Primer5.0 by referring to the sequences provided by Genbank (RNA fragment sequences are shown in **Table 2**). The procedure was conducted as follows: 1st cycle 95°C for 2 min, followed by denaturation at 95°C for 5 s, annealing at 60°C for 10 s, extension at 72°C for 30 s, 40 cycles, and finally terminated at 72°C for 5 min. The Ct value was the number of cycles the fluorescence signal in each reaction tube undergoes when it reaches a set threshold value. The specificity of the reaction products was determined based on the solubility curve and the results of product electrophoresis. The relative ratio of the target gene to GAPDH was used as its expression, and the relative ratio was calculated using the 2-△△Ct method.

**Table 2:** PCR primers.

Gene	Forward primer sequence	Reverse primer sequence
NR4A2	TTACGCTACCACCGGAGTTC	AGAAGTGAGTAGAAGCGGCG
GAPDH	GACATGCCGCCTGGAAAAC	AGCCCAGGATGCCCTTTAGT

### Statistical analysis

R (version 4.1.3) was utilized for statistical analysis. A p-value < 0.05 was set as a significance threshold for screening DEGs, immune infiltration analysis, immune-related gene model, QPCR, WB experimental results data analysis, and drug prediction.

## Results

### Identification of DEGs

**Figure 2:**
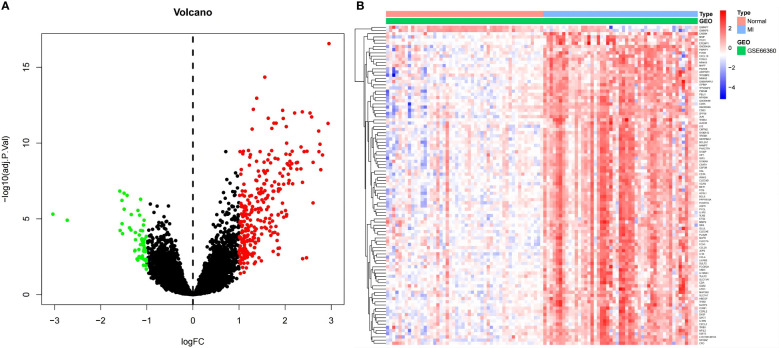
**(A)** Volcano plot for DEGs. Red dots indicate upregulated DEGs, and green dots express downregulated DEGs. **(B)** Heatmap for DEGs. Each line symbolizes one DEG, and each row represents one specimen. The red and blue swatches represent upregulated and downregulated DEGs, respectively.

Dataset GSE66360 was selected and underwent differential expression analysis using the “Limma” package in R 4.1.3 software. Three hundred thirty-five DEGs were identified, including 280 up- and 55 downregulated genes (log2FC > 1 and P-value <0.05). These DEGs were visualized in a volcano plot (**Figure 2A**) with downregulated genes in green, upregulated genes in red, and the rest in black. The top 50 upregulated and downregulated genes were selected to create a heatmap (**Figure 2B**).

### Single-cell RNA sequencing analysis

**Figure 3:**
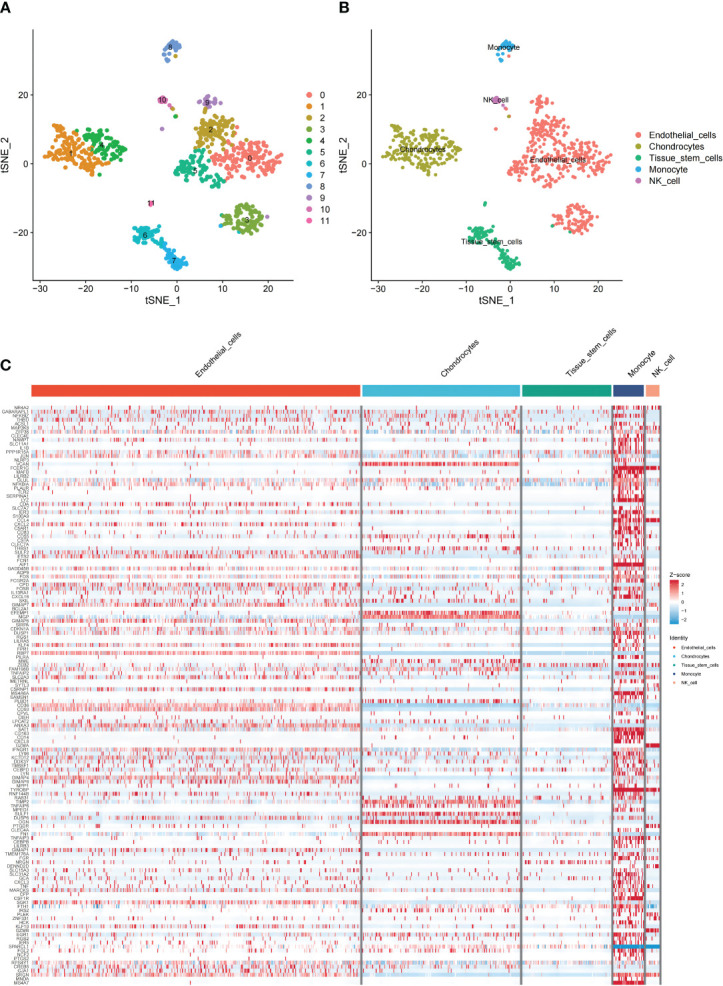
Overview of single cells from GSE180678. **(A)** Clusters of 12 cells embedded in a T-distributed Stochastic Neighbor Embedding (tSNE). **(B)** Marker genes were used to identify the cell types. **(C)** Heat map of the distribution and enrichment of DEGs in cell subpopulations.

Cluster analysis categorized scRNA-seq cells into 12 clusters using the “FindNeighbors” and “FindCluster” functions (**Figure 3A**). Marker genes were used to categorize the 12 clusters into 5 types of cells (**Figure 3B**). Next, the “FindAllMarkers” function was used to screen the DEGs and 144 genes intersected with the sequencing results. We found that the DEGs were predominantly distributed in endothelial cells. This implies that endothelial cells may have an important role in MI (**Figure 3C**).

### DO enrichment analysis

**Figure 4:**
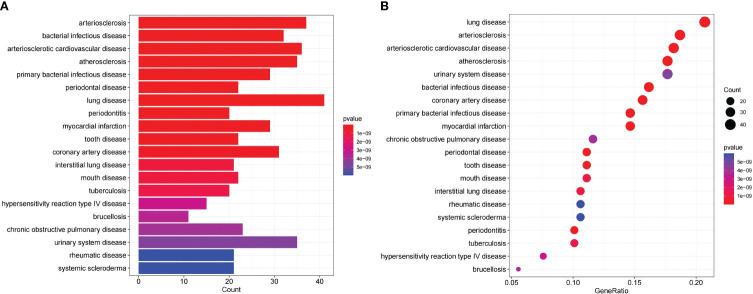
DO enrichment analysis shows the correlation between MI and the DEGs; **(A)** The x-axis of the bar chart displays the number of genes, and the y-axis displays the correlative diseases. **(B)** The x-axis of the bubble plot shows the ratio of the genes, and the y-axis shows the correlative diseases.

We conducted an enrichment analysis of significant genes and diseases to observe the enrichment degree of DEGs and MI. As shown in **Figure 4**, DEGs were significantly enriched in diseases such as arteriosclerosis, bacterial infectious disease, arteriosclerotic cardiovascular disease, atherosclerosis, primary bacterial infectious disease, periodontal disease, lung disease, periodontitis, and myocardial infarction. 29 genes were significantly enriched in MI, suggesting a correlation between these DEGs and MI, highlighting the robust correlation between DEGs and MI. (For more information, please see **Supplementary Chart 1**).

### Immune infiltration analysis

**Figure 5:**
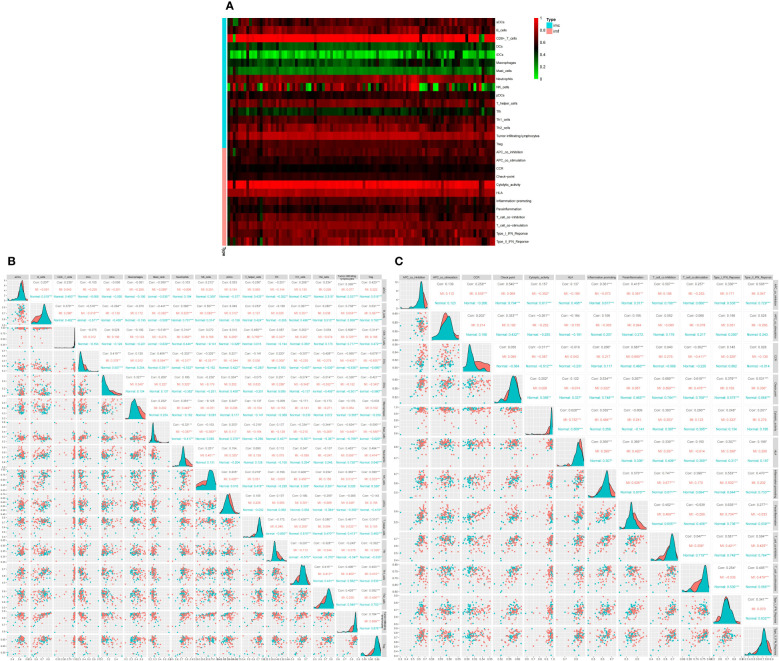
Differential expression of immune score between normal and MI groups, * indicates the significance of the correlation and the number indicates the degree of correlation. **(A)** Heatmap for DEGs. Each line represents one immune cell or function, and each column represents one dataset. Genes with high correlation and no correlation were represented in red and green swatches, respectively.The redder the color of each row, the higher the correlation. Imc indicates immune cells and imf indicates immune function **(B)** Correlation matrix of 16 significant immune cell subtypes. **(C)** Correlation matrix of 12 immune function subtypes. * indicates the significance of the correlation and the number indicates the degree of correlation (*P<0.05, **P<0.01, ***P<0.001).

Subsequently, we conducted ssGSEA to appraise immune cell content and function in DEGs. The results showed that the top three immune cells with the highest content of DEGs were CD8+T cells, B cells, and Neutrophils, while the highest immune score was found in cytolytic activity (**Figure 5A**).Then, we analyzed the immune infiltration of immunological cells and functions. The corrplot of immune infiltration divergence illustrated that the levels of 16 immune cells and 12 types of immune functions were high in MI patients.

Analysis of 16 immune cell types revealed that B cells were positively linked to TILs (r=0.738), regulatory T cells (Tregs) (r=0.631), and Natural killer (NK) cells (r=0.557). TILs were positively correlated to B cells, CD8+T cells (r=0.696), and Tregs (r=0.784), while Tregs were positively correlated with TILs, B cells, and Th2 cells (r=0.582). On the other hand, mast cells were negatively correlated with Tregs (r =-0.599), B cells (r=-0.441), TIL (r =-0.624), and CD8+T cells (r=-0.518). Moreover, Dendritic cells (DCs) were negatively correlated to B cells (r =-0.516), Tregs (r=-0.678), and TILs (r =-0.565) (**Figure 5B**). The corrplot of the immune function divergence indicated that parainflammation was positively linked to C-C chemokine receptor (CCR) (r=0.687), inflammation-promotion (r=0.57), and type I interferon (IFN) response (r=0.635), while T cell co-inhibition was positively associated with APC co-inhibition (r=0.507), checkpoints (r=0.68), inflammation-promotion(r=0.747) and T cell co-stimulation (r=0.547), type I IFN response (r=0.581), and type II IFN response (r=0.594) (**Figure 5C**).

### Differential analysis of immune scores between MI and control groups

**Figure 6:**
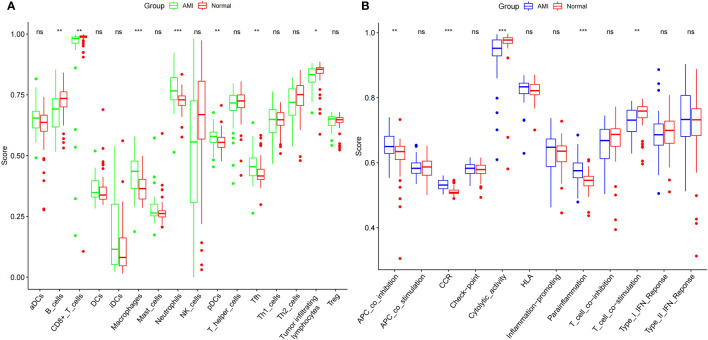
Comparison of expression of immune cells **(A)** and functions **(B)** between the MI patients and controls. *** indicates that the P-value is < 0.001, ** indicates that the P-value is < 0.01 * indicates P < 0.05, and “ns” indicates P < 1.

After integrating the data of the MI and normal groups from the two datasets, we analyzed the difference in immune cells and immune function. We found that the Cell types that show the most difference in immune cell infiltration between MI and control are macrophages and neutrophils (**Figure 6A**). We also found a significant difference among the seven immune functions, with the most significant difference observed in CCR, Cytolytic activity, Parainflammation, followed by APC co-inhibition, and T cell co-stimulation. This phenomenon suggests that these immune functions may be involved at critical times in the pathophysiology of MI (**Figure 6B**).

### Relevance analysis for immune cells and functions from DEGs

**Figure 7:**
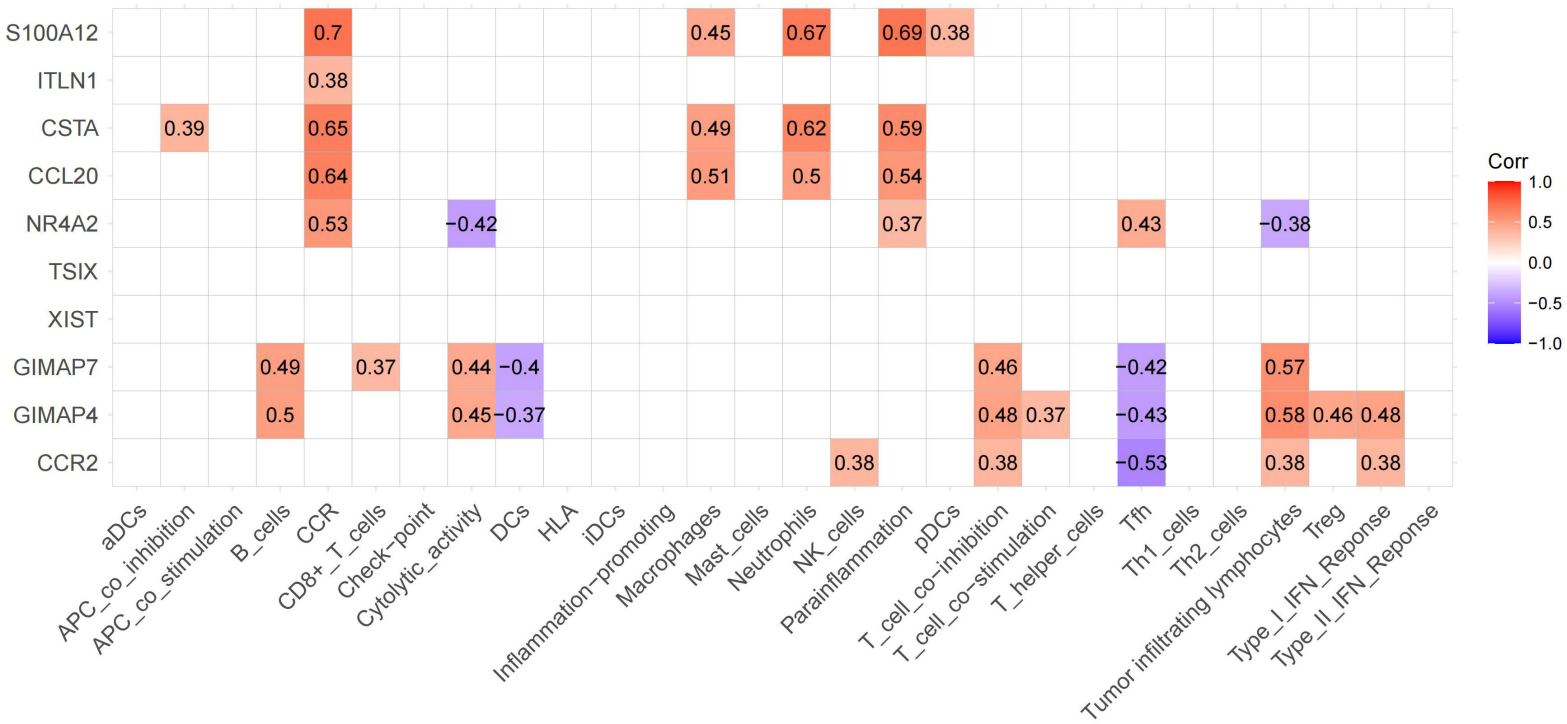
Expression of significant genes associated with immune functions. The upregulated and downregulated correlations are displayed in red and purple, respectively. We labeled these 6 genes as hub genes.

The top 5 upregulated and downregulated genes from DEGs were used to analyze the association between immune cells and functions (**Figure 7**). We found the highest positive correlation between S100A12 vs. CCR, followed by S100A12 vs. Parainflammation and S100A12 vs. Neutrophils. Conversely, the highest negative correlation was found between CCR2 vs. Tfh, followed by GIMAP4 vs. Tfh. The highest correlation between immune cells and function was observed in 6 genes: GIMAP7, GIMAP4, CCR2, NR4A2, CSTA, and S100A12.

### Immune-gene model for diagnosis of MI

**Figure 8:**
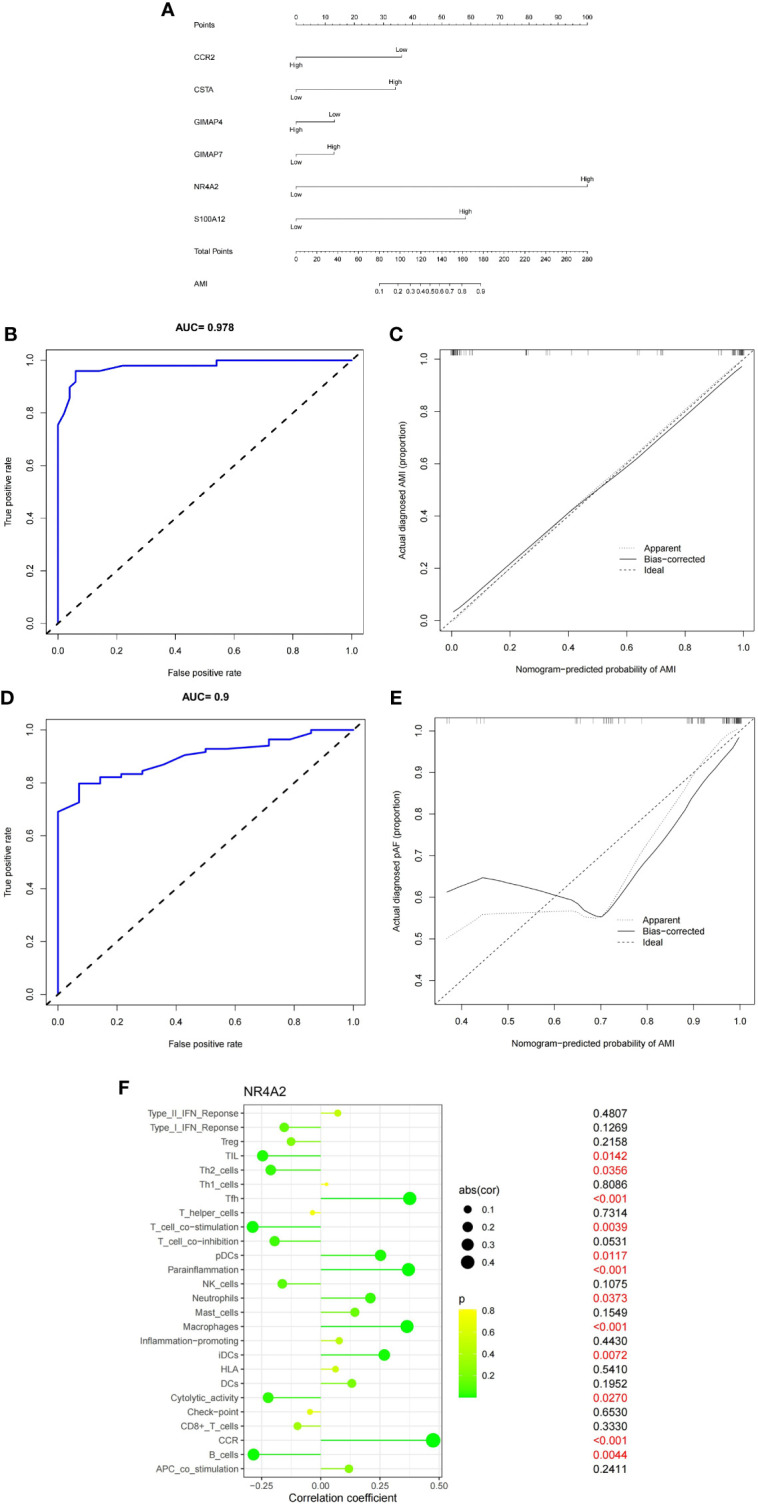
**(A)** Six genes (CCR2, P=0.07;CSTA, P=0.18; GIMAP4, P=0.69; GIMAP7, P=0.71; NR4A2, P=0.0004; S100A12, P=0.04) were utilized to establish a nomogram for the diagnosis MI. **(B)** ROC curves of the nomogram. **(C)** Calibration chart of the diagnostic model. **(D)** ROC curves of the nomogram in the validation set. **(E)** Calibration plots for validation sets. **(F)** Correlation of NR4A2 with infiltrating immune cells in MI and normal samples.

We used hub genes to build gene-disease diagnostic models and generated a line chart, calibration map, and AUC curve. The nomogram was utilized to visualize the model, including the 6 hub genes. The fraction of each gene was consistent with the proportion in the nomogram. As shown in the nomogram (**Figure 8A**), GIMAP7, GIMAP4, CCR2, NR4A2, CSTA, and S100A12 were predicting factors of MI. High levels of NR4A2 and S100A12 were positively associated with the diagnosis of MI. Hence, the accuracy of MI diagnosis was predicted based on the total score of the three characteristics. The model’s performance was appraised by C-index, AUC, and calibration plots. The ROC curve of the model yielded an AUC value of 0.978, indicating that the model had good discrimination ability (**Figure 8B**). The calibration plot also indicated the better correction of the model (**Figure 8C**). To validate the accuracy of the prediction model, we performed a validation using a test dataset. The accuracy was evaluated by AUC plots. Interestingly, the AUC value (**Figure 8D**) and calibration plot (**Figure 8E**) was set at 0.9, indicating that the accuracy of the HUB gene is more reliable for the diagnosis of MI. Finally, we further explored the correlation between NR4A2 expression and immune cells (**Figure 8F**). As displayed in **Figure 8F**, NR4A2 was positively correlated with Tfh, pDCs, Parainflammation, Neutrophils, Macrophages, iDCs, and CCR. and negatively correlated with Tumor Infiltrating Lymphocytes, Th2 cells, T cell co−stimulation, Cytolytic activity, and B cells.

### Drug prediction analysis

**Figure 9:**
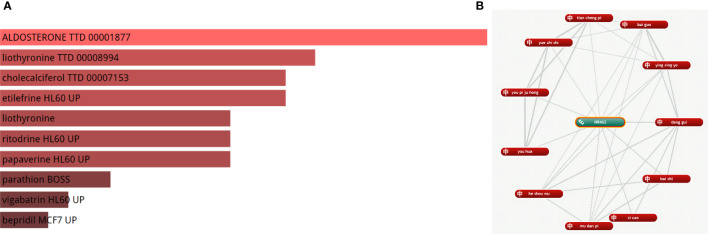
**(A)** The top 10 medicines for MI. **(B)** The 11 Chinese herbal medicines for MI.

We identified a strong correlation between 180 drugs and MI. Finally, the top five drugs Aldosterone, liothyronine, cholecalciferol, etilefrine, and liothyronine, were selected (adjusted P-value < 0.05) (**Figure 9A**), (For more information, please see **Supplementary Chart 2**). Meanwhile, we found that 11 herbal medicines were associated with NR4A2 (**Figure 9B**); the top 6 herbal medicines were danggui, baizhi, zicao, mudanpi, heshouwu, and youhua.

### Quantitative real-time PCR and Western blot analysis

**Figure 10:**
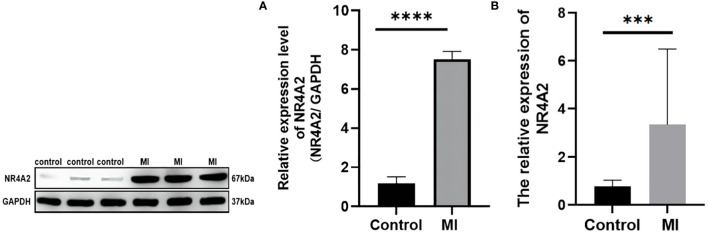
qPCR and Western blotting analysis of controls vs. MI rats. **(A)** Western blot analysis. There were significant differences in NR4A2 protein levels between controls and MI rats (p < 0.0001). **(B)** mRNA level of NR4A2 in controls vs. MI (p<0.001). *** indicates P < 0.001, **** indicates P < 0.0001.

The transcriptional changes of NR4A2 in the heart tissues of MI rats and controls were detected by qRT-PCR and WB. The results showed that the expression of NR4A2 was increased in both MI groups compared with the control group (**Figures 10A, B**), which was consistent with the in silico analysis results and indicated the potential diagnostic value of NR4A2 for MI.

## Discussion

MI is the most severe cardiovascular disease worldwide, but there is a clinical lag in diagnosing MI, accounting for the high mortality risk from MI in developing and developed countries. Immunological represents a landmark in the history of medicine. As modern research progresses, there is increasing evidence that immune activation and suppression play important and influential roles in many diseases. However, while inflammation and oxidative stress have been predominantly investigated in the cardiovascular field, immune pathways have been largely underexplored, with few studies assessing whether immune-related genes are of value in diagnosing MI. Therefore, our research used ssGSEA to assess the average expression levels of 16 immune cell types and 12 immune functions in the MI population. Finally, the DEGs were integrated with immune scores to screen the top six genes with the most significant correlation with MI for gene-disease diagnosis. In this study, we constructed a nomogram for the diagnosis of MI based on immune cells and function. As a result, we identified NR4A2 as an independent predictor of MI.

Interestingly, immunity can directly or indirectly regulate the inflammatory response in the body. Inflammation is the initial response of cardiomyocytes after infarction (23), and inflammatory factors activate adaptive immune pathways *in vivo*, which in turn promote the reaction of immune cells in cardiomyocytes in the infarct area (24). At the same time, immune infiltration of cardiomyocytes has a bidirectional regulatory role. Overwhelming evidence suggests that immune infiltration of cardiomyocytes exacerbates the inflammatory response (25). It should be borne in mind that immune activation can also repair damaged cardiomyocytes. Herein, we substantiated that macrophages and neutrophils are most abundant in the MI population than in the general population. Based on extensive fundamental research, we found that activation of immune cells, especially macrophages and neutrophils, participate in critical regulatory mechanisms for sterile inflammation *in vivo (* 26).

During the inflammatory phase of infarction, inflammatory factors activate macrophages and neutrophils (27). Activated macrophages secrete anti-inflammatory cytokines (28–30), which reduce thrombosis and thus protect the heart (31). After infarction, activated macrophages gradually fill the infarct site by downregulating inflammatory cytokines and upregulating inflammatory factors such as IL-10, VEGF, and TGF-β (32–35), establishing an anti-inflammatory environment that protects cardiomyocytes in the infarct area from apoptosis and maintains cell survival function. In addition, glucocorticoid receptors in macrophages play a crucial role in post-infarct myocardial repair by regulating the differentiation of myofibroblasts in the infarct microenvironment (36, 37). Conversely, activated neutrophils lead to the release of neutrophil myeloperoxidase (38), which can accelerate protease-mediated degradation of basement membrane components and endothelial damage (39), promoting atherosclerotic plaque damage (40) as well as plaque instability. Importantly, atherosclerotic plaques become more vulnerable in the proteolytic environment of the basement membrane and core (41), leading to plaque fragility and rupture (42) and increasing the risk of myocardial infarction.

In the present study, we found that the expression of these three immune functions differed significantly between healthy and MI populations. CCR and Parainflammation were significantly upregulated, while Cytolytic activity was downregulated in the MI population. The mechanism may be related to the activation of immune function due to massive inflammatory infiltration in cardiomyocytes. There is a rich literature available suggesting that the recruitment of monocytes and monocyte-derived macrophages is the hallmark response to cardiomyocyte death and tissue-resident cardiac CCR2+ macrophages are important upstream mediators of the inflammatory response to myocardial injury (43). Activation of CCR2+ macrophages leads to the upregulation of inflammatory chemokines and cytoplasmic inflammatory factors (44), exacerbating the apoptosis of cardiomyocytes after myocardial infarction (45).

Parainflammation in cardiovascular disease (CVD) is associated with low and persistent circulating levels of pro-inflammatory proteins (46). In CVD, vascular integrity may be disrupted by pro-inflammatory cytokines that promote macrophage infiltration through the vessel wall to form atherosclerotic plaques and increase susceptibility to plaque instability in later stages (47). On the contrary, Cytolytic cells usually induce apoptosis of abnormal cells. In states of infection or inflammation, lysis induces apoptosis of activated macrophages and T cells to control the inflammatory response (48) to ensure plaque stability and delay the onset of MI. Thus, the immunoassay results in this study are consistent with the literature, emphasizing these cells’ importance in the pathogenesis of MI and providing the basis for our subsequent analysis.

Undeniably, immune pathways play critical regulatory mechanisms in the pathogenesis of MI. However, the study of immune processes remains the domain of fundamental theoretical research. Whether immune-related genes can be applied in the clinic warrants further investigation. After immune analysis of MI-related genes, we found that NR4A2 has potential clinical diagnostic value for MI. Our study found that the amount of NR4A2 was positively correlated with MI. The mechanism may be related to the release of pro-inflammatory factors *in vivo* under immune regulation after myocardial infarction (49). However, further studies are warranted to corroborate the relationship between NR4A2 and immune activation. Previous studies found that activation of NR4A2 was an adaptive response to ischemia-induced apoptosis in cardiomyocytes (50). Interestingly, cardiomyocytes after myocardial infarction undergo significant apoptosis due to ischemia (51). When ischemia persists, cells within the infarcted region initiate autophagic mechanisms to slow the progression of cardiomyocyte death (52). A cellular study (50) showed a significant increase in NR4A2 expression in H9C2 cardiomyocytes after ischemia, especially when the ischemic time exceeded 4 h. Consistently, we demonstrated that NR4A2 significantly reduced cardiomyocyte apoptosis, thereby protecting the survival of cardiomyocytes after infarction. Knockdown of NR4A2 blocked the autophagic flow of cardiomyocytes and promoted apoptosis, possibly through a significant increase in P53 expression after NR4A2 low presentation, thereby inhibiting the autophagic response of cardiomyocytes in the infarct region (50). In summary, we conclude that NR4A2 has diagnostic value for MI for the bioinformatics and experimental validation of MI.

Nevertheless, there are some limitations in this study. First, the accuracy of the diagnostic model may be biased due to the limited database resources and sample size. Besides, our drug prediction results have no experimental basis. Accordingly, the results of this study should be interpreted with caution and validated in future studies *via in vivo* and *in vitro* experiments. Finally, our experiments are based on *in vivo* experiments in animals, and this study can only tentatively explain that NR4A2 has potential diagnostic value. However, as clinical studies in humans were not performed in this study, it cannot be asserted that NR4A2 has a definite diagnostic accuracy for MI. The conclusions drawn from this study should be interpreted with caution.

## Conclusion

In summary, we evaluated the immune infiltration patterns in MI based on GEO data from ssGSEA and constructed a nomogram containing 6 HUB(GIMAP7, GIMAP4, CCR2, NR4A2, CSTA, and S100A12) genes for the diagnosis of MI. Applying single-cell sequencing, DO enrichment analysis, and drug prediction analysis may provide a new direction for studying the relationship between myocardial infarction, immune response, and efficacy.

## Data availability statement

Publicly available datasets were analyzed in this study. This data can be found here: https://www.ncbi.nlm.nih.gov/geo/. The accession number(s) can be found in the article.

## Ethics statement

The animal study was reviewed and approved by Ethics Committee of the Affiliated Hospital of Liaoning University of Traditional Chinese Medicine.

## Author contributions

DW analyzed and wrote the manuscript. JQ designed the experiments and analyzed the data. LL, YW and XH participated in the qPCR and WB experiments, ZZ and GY designed and supervised the study, and did the final review and revision of the article before submission.
