# Supplementary figures and images for: NR4A2 may be a potential diagnostic biomarker for myocardial infarction: A comprehensive bioinformatics analysis and experimental validation
Source: Front Immunol. 2022 Dec 22;13:1061800. doi: 10.3389/fimmu.2022.1061800 (PMC9815548; doi:10.3389/fimmu.2022.1061800)

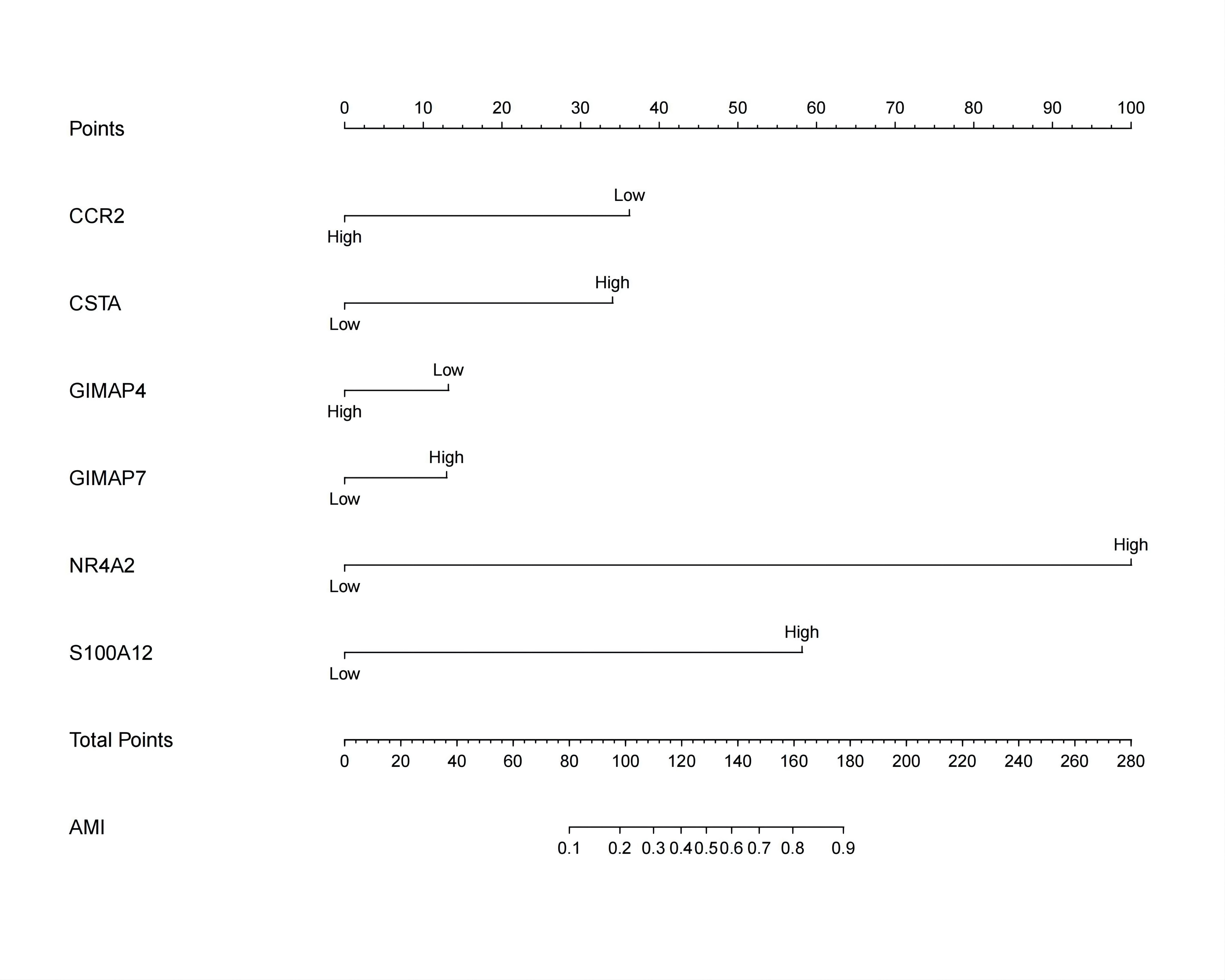

Supplement: Supplementary file 1 [file Image_1.tif]

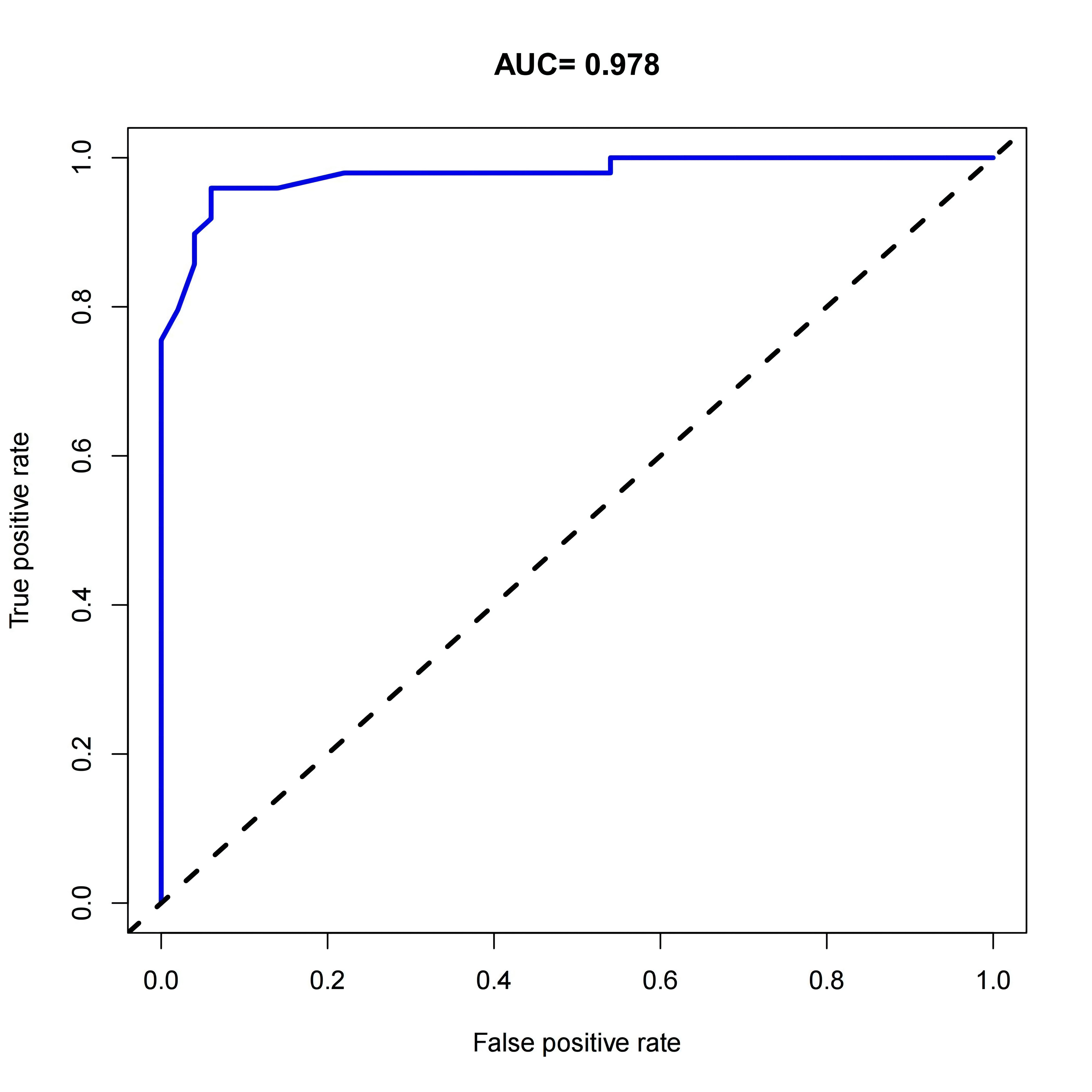

Supplement: Supplementary file 2 [file Image_2.tif]

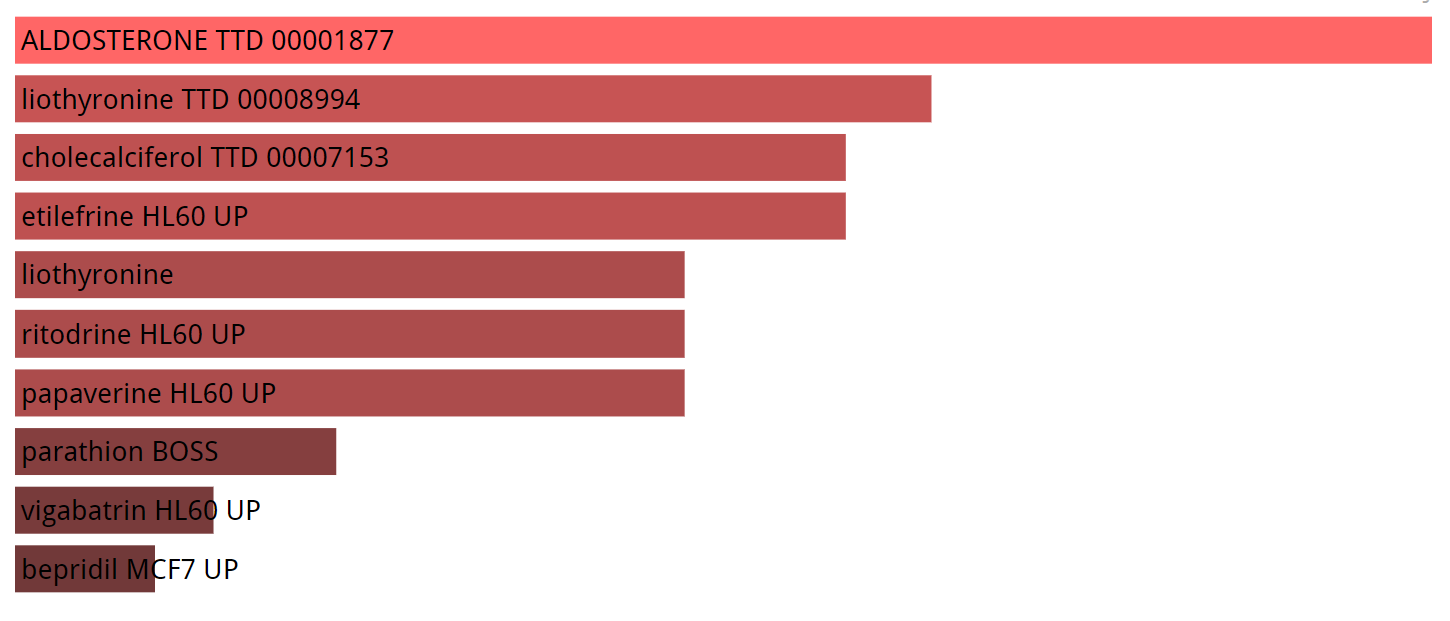

Supplement: Supplementary file 3 [file Image_3.tif]

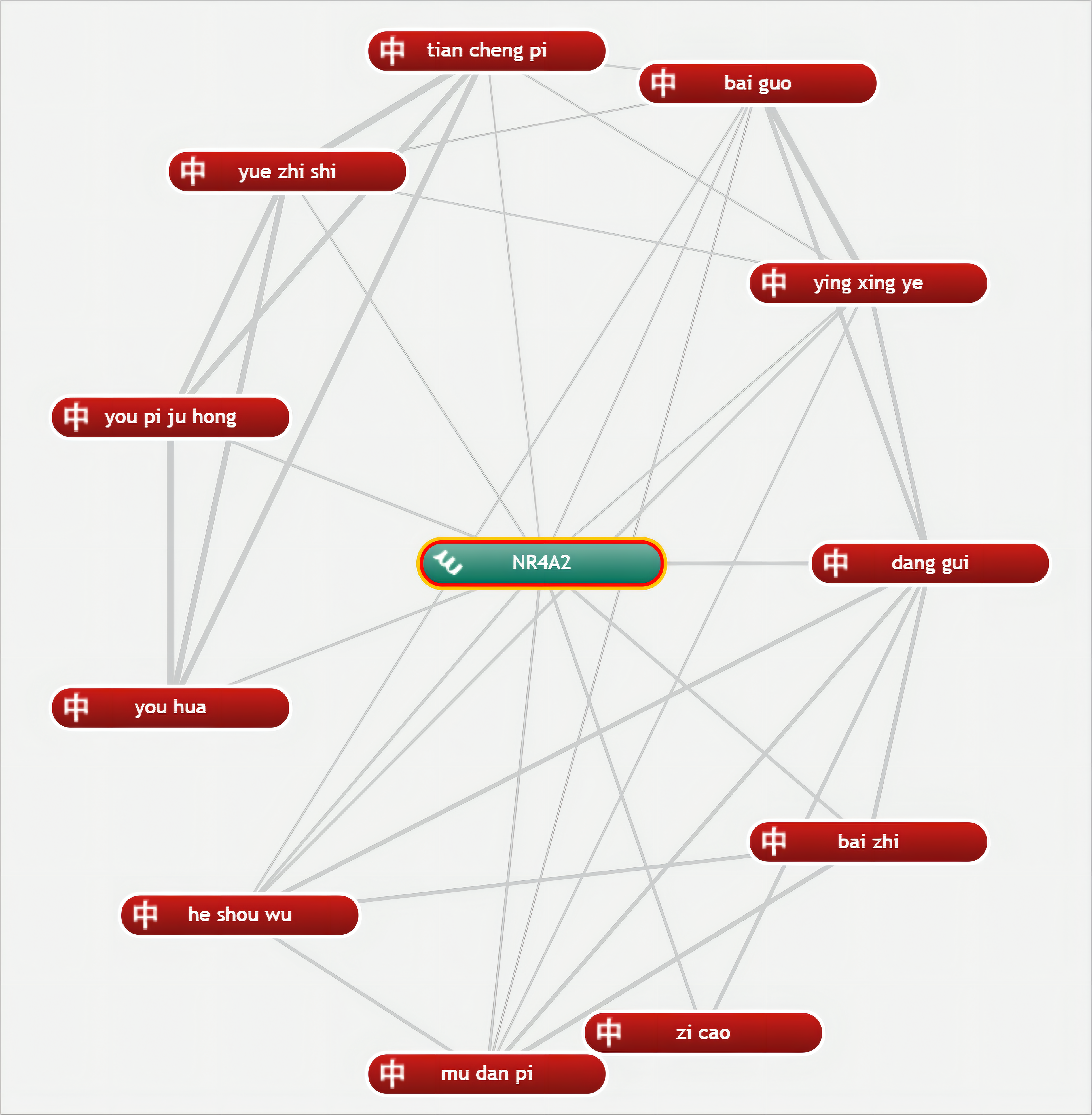

Supplement: Supplementary file 4 [file Image_4.tif]

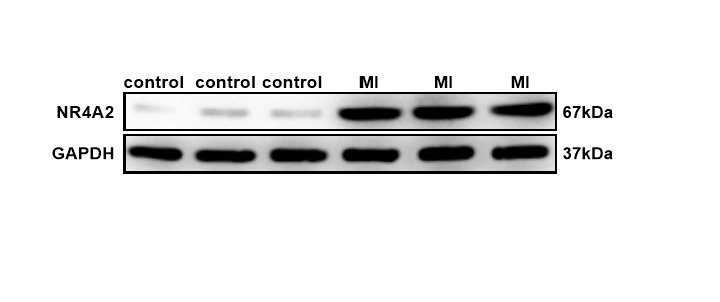

Supplement: Supplementary file 5 [file Image_5.tif]

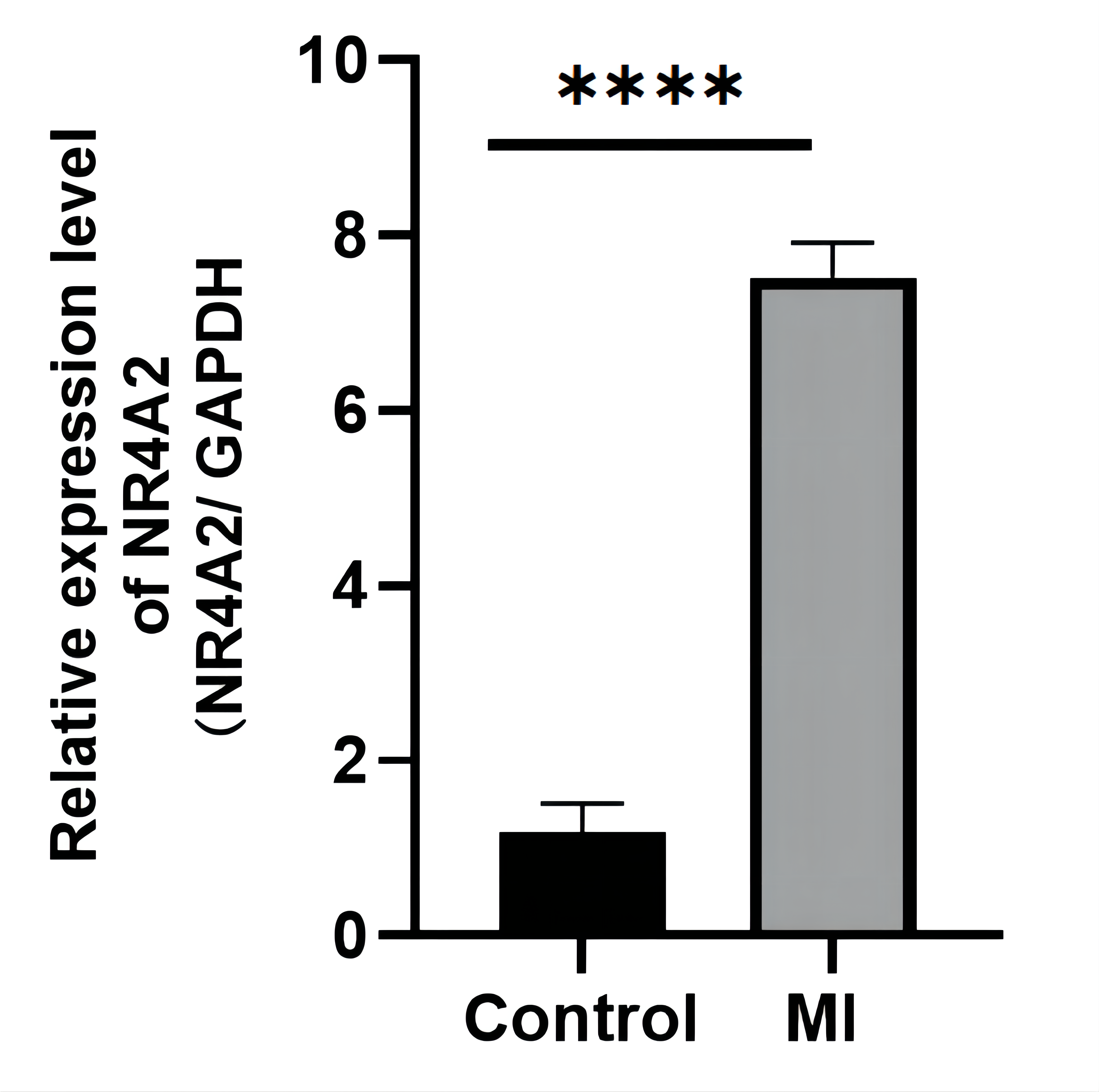

Supplement: Supplementary file 6 [file Image_6.tif]

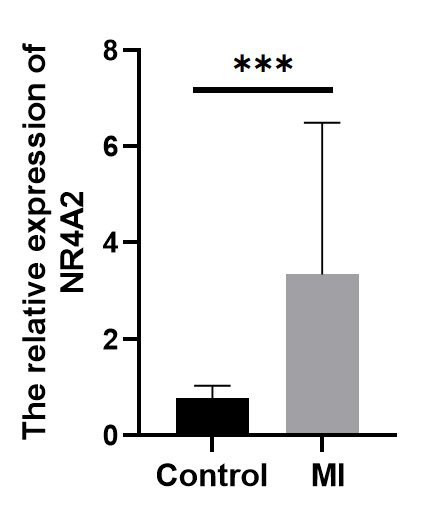

Supplement: Supplementary file 7 [file Image_7.tif]

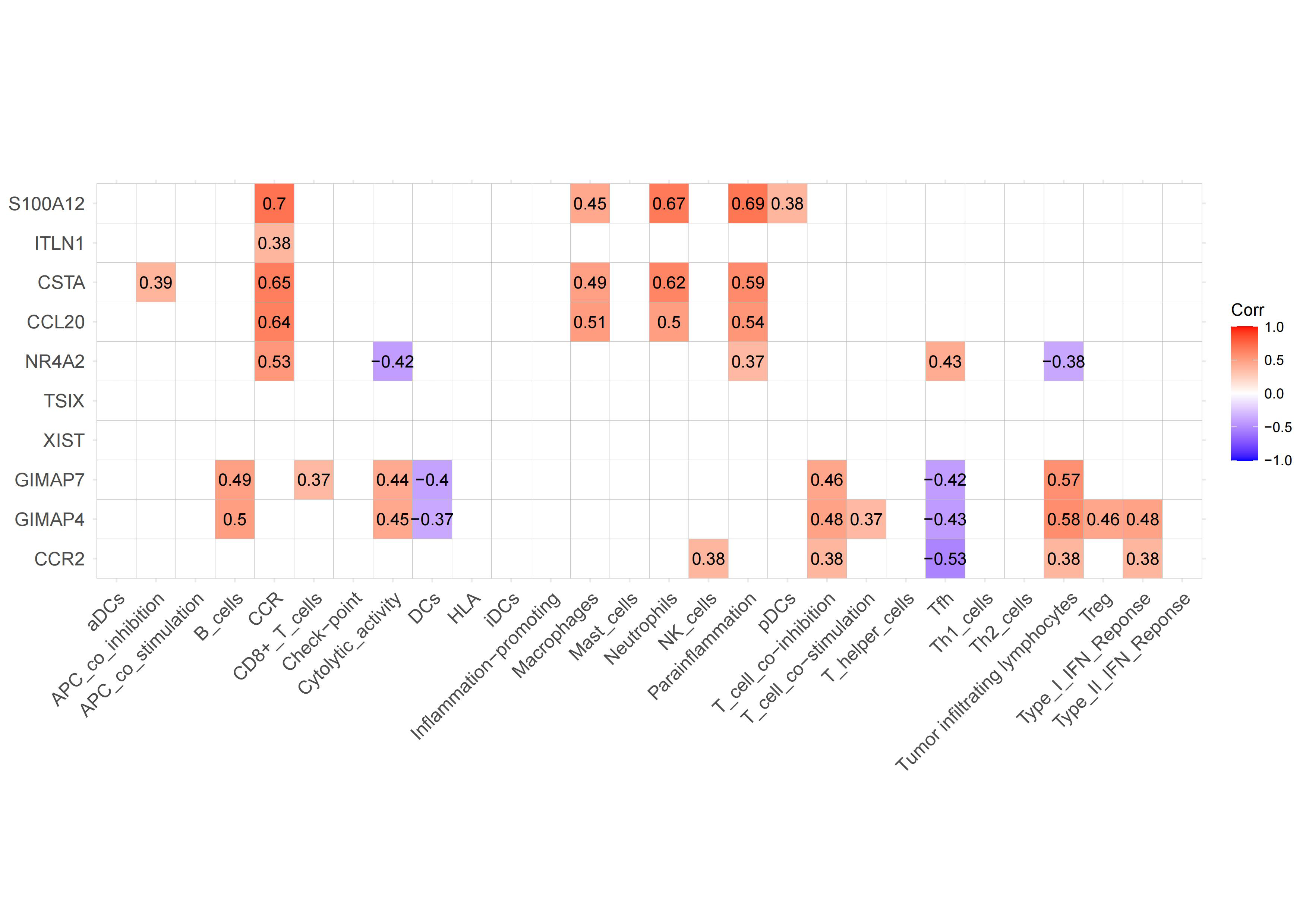

Supplement: Supplementary file 8 [file Image_8.tif]
