# Supplementary material for: NR4A2 may be a potential diagnostic biomarker for myocardial infarction: A comprehensive bioinformatics analysis and experimental validation
Source: Front Immunol. 2022 Dec 22;13:1061800. doi: 10.3389/fimmu.2022.1061800 (PMC9815548; doi:10.3389/fimmu.2022.1061800)
Supplement: Supplementary file 9 [file Table_1.docx]

Supplementary Material

# Supplementary Figures and Tables

Supplementary Table 1:DO enrichment analysis of DEGs

| ID | Description | GeneRatio | BgRatio | pvalue | p.adjust | Qvalue | geneID | Count |
| --- | --- | --- | --- | --- | --- | --- | --- | --- |
| DOID:2349 | arteriosclerosis | 37/198 | 356/8007 | 4.44E-14 | 2.17E-11 | 1.03E-11 | THBD/S100A12/NAMPT/IL1B/HBEGF/NLRP3/PLAUR/IL1RN/TLR2/LYZ/S100A9/AIF1/ALDH2/ICAM1/FCGR2A/CXCL16/MGP/MMP9/FPR1/CCR2/PTX3/CCR5/TLR4/CD36/CXCL1/CD163/CD14/CHI3L1/ADM/S100A8/EDN1/FN1/TNF/LGALS2/PLA2G7/PTGS2/MNDA | 37 |
| DOID:104 | bacterial infectious disease | 32/198 | 271/8007 | 8.38E-14 | 2.17E-11 | 1.03E-11 | THBD/SLC11A1/IL1B/CCL20/JUN/HBEGF/NLRP3/PLAUR/IL1RN/TLR2/TREM1/CCL4/C5AR1/ICAM1/FCGR2A/CXCL16/BCL2A1/MMP9/PTX3/CCR5/FPR2/TLR4/PTAFR/CISH/CD14/CXCL8/GZMA/IFNGR1/LY96/TNF/PTGS2/SLC22A4 | 32 |
| DOID:2348 | arteriosclerotic cardiovascular disease | 36/198 | 345/8007 | 9.13E-14 | 2.17E-11 | 1.03E-11 | THBD/S100A12/NAMPT/IL1B/HBEGF/NLRP3/PLAUR/IL1RN/TLR2/LYZ/S100A9/ALDH2/ICAM1/FCGR2A/CXCL16/MGP/MMP9/FPR1/CCR2/PTX3/CCR5/TLR4/CD36/CXCL1/CD163/CD14/CHI3L1/ADM/S100A8/EDN1/FN1/TNF/LGALS2/PLA2G7/PTGS2/MNDA | 36 |
| DOID:1936 | atherosclerosis | 35/198 | 344/8007 | 4.51E-13 | 8.03E-11 | 3.82E-11 | THBD/S100A12/NAMPT/IL1B/HBEGF/NLRP3/PLAUR/IL1RN/TLR2/S100A9/ALDH2/ICAM1/FCGR2A/CXCL16/MGP/MMP9/FPR1/CCR2/PTX3/CCR5/TLR4/CD36/CXCL1/CD163/CD14/CHI3L1/ADM/S100A8/EDN1/FN1/TNF/LGALS2/PLA2G7/PTGS2/MNDA | 35 |
| DOID:0050338 | primary bacterial infectious disease | 29/198 | 238/8007 | 6.21E-13 | 8.84E-11 | 4.21E-11 | THBD/SLC11A1/IL1B/CCL20/JUN/HBEGF/NLRP3/PLAUR/IL1RN/TLR2/TREM1/CCL4/C5AR1/ICAM1/FCGR2A/BCL2A1/MMP9/PTX3/CCR5/TLR4/CISH/CD14/CXCL8/GZMA/IFNGR1/LY96/TNF/PTGS2/SLC22A4 | 29 |
| DOID:3388 | periodontal disease | 22/198 | 139/8007 | 2.41E-12 | 2.86E-10 | 1.36E-10 | IL1R2/IL1B/CCL20/IL1RN/TLR2/LYZ/ALDH2/FCGR2A/MMP9/FPR1/CCR2/CCR5/TLR4/CD14/CXCL8/FCGR3B/ADM/S100A8/EDN1/CEBPB/TNF/PTGS2 | 22 |
| DOID:850 | lung disease | 41/198 | 499/8007 | 4.26E-12 | 4.33E-10 | 2.06E-10 | THBD/IRAK3/SLC11A1/IL1B/PPP1R15A/FOSL2/NFKBIA/PLAUR/IL1RN/TLR2/SERPINA1/TREM1/S100A9/C5AR1/CSTA/ICAM1/FOS/FCGR2A/IL13RA1/MMP9/CCR2/PTX3/CCR5/TLR4/CD36/RNASE2/CD14/CXCL8/GZMA/IFNGR1/FCGR3B/CHI3L1/ADM/EDN1/TNF/SGK1/HCK/CSF2RB/FGL2/NCF2/PTGS2 | 41 |
| DOID:824 | periodontitis | 20/198 | 120/8007 | 9.50E-12 | 8.45E-10 | 4.02E-10 | IL1R2/IL1B/IL1RN/TLR2/LYZ/ALDH2/FCGR2A/MMP9/FPR1/CCR2/CCR5/TLR4/CD14/CXCL8/FCGR3B/ADM/S100A8/CEBPB/TNF/PTGS2 | 20 |
| DOID:5844 | myocardial infarction | 29/198 | 279/8007 | 3.43E-11 | 2.72E-09 | 1.29E-09 | THBD/IL1B/PLAUR/IL1RN/TLR2/S100A9/THBS1/TRIB1/ALDH2/ICAM1/FCGR2A/MMP9/CCR2/PTX3/CCR5/TLR4/CD36/CD163/CD14/CXCL8/ADM/EDN1/FN1/TNF/LGALS2/PLA2G7/GZMB/PTGS2/SERPINB2 | 29 |

Supplementary Table 2:Drug prediction

| Term | Overlap | P-value | Adjusted P-value | Old P-value | Old Adjusted P-value | Odds Ratio | Combined Score | Genes |
| --- | --- | --- | --- | --- | --- | --- | --- | --- |
| ALDOSTERONE TTD 00001877 | 1月16日 | 8.00E-04 | 0.01673977 | 0 | 0 | 19984 | 142504.3668 | NR4A2 |
| liothyronine TTD 00008994 | 1月21日 | 0.001049976 | 0.01673977 | 0 | 0 | 19979 | 137035.7122 | NR4A2 |
| cholecalciferol TTD 00007153 | 1月22日 | 0.001099976 | 0.01673977 | 0 | 0 | 19978 | 136099.4703 | NR4A2 |
| etilefrine HL60 UP | 1月22日 | 0.001099976 | 0.01673977 | 0 | 0 | 19978 | 136099.4703 | NR4A2 |
| liothyronine | 1月24日 | 0.001199974 | 0.01673977 | 0 | 0 | 19976 | 134347.6949 | NR4A2 |
| ritodrine HL60 UP | 1月24日 | 0.001199974 | 0.01673977 | 0 | 0 | 19976 | 134347.6949 | NR4A2 |
| papaverine HL60 UP | 1月24日 | 0.001199974 | 0.01673977 | 0 | 0 | 19976 | 134347.6949 | NR4A2 |
| parathion BOSS | 1月29日 | 0.00144997 | 0.01673977 | 0 | 0 | 19971 | 130534.6917 | NR4A2 |
| vigabatrin HL60 UP | 1月31日 | 0.001549969 | 0.01673977 | 0 | 0 | 19969 | 129189.851 | NR4A2 |
| bepridil MCF7 UP | 1/32 | 0.001599968 | 0.01673977 | 0 | 0 | 19968 | 128549.4196 | NR4A2 |
